# Lipopolysaccharide, VE-cadherin, HMGB1, and HIF-1α levels are elevated in the systemic circulation in chronic migraine patients with medication overuse headache: evidence of leaky gut and inflammation

**DOI:** 10.1186/s10194-024-01730-5

**Published:** 2024-02-19

**Authors:** Doga Vuralli, Merve Ceren Akgor, Hale Gok Dagidir, Ozlem Gulbahar, Meltem Yalinay, Hayrunnisa Bolay

**Affiliations:** 1 Department of Neurology and Algology, Gazi University Faculty of Medicine, Ankara, Turkey; 2 Neuroscience and Neurotechnology Center of Excellence (NÖROM), Gazi University, Ankara, Turkey; 3 Neuropsychiatry Center, Gazi University, Ankara, Turkey; 4 Department of Medical Biochemistry, Gazi University Faculty of Medicine, Ankara, Turkey; 5 Department of Clinical Microbiology, Gazi University Faculty of Medicine, Ankara, Turkey

**Keywords:** Medication overuse headache, Chronic migraine, Lipopolysaccharide, Lipopolysaccharide-binding protein, Leaky gut, HMGB1, HIF-1α, Occludin, VE-Cadherin, Inflammation

## Abstract

**Objective:**

Medication overuse headache (MOH) was recently shown to be associated with leaky gut in rodents. We aimed to investigate whether chronic migraine (CM) patients with MOH have elevated lipopolysaccharide levels and inflammatory molecules in blood circulation.

**Materials and methods:**

The study included women participants (40 CM patients with NSAID overuse headache, 35 episodic migraine (EM) patients, and 20 healthy non-headache sufferers). Migraine duration, monthly migraine headache days, MigSCog, HADS-D, HADS-A, and HIT-6 scores were recorded. Serum samples were collected to measure circulating LPS, LPS binding protein (LBP), tight junction protein occludin, adherens junction protein vascular endothelial cadherin (VE-cadherin), CGRP, HMGB1, HIF-1α, IL-6, and IL-17 levels.

**Results:**

Serum LPS, VE-Cadherin, CGRP, HIF-1α, and IL-6 levels were significantly higher in the CM + MOH group compared to the EM group and healthy controls while serum LBP and HMGB1 were higher in the CM + MOH group compared to healthy controls. IL-17 and occludin levels were comparable between the three groups. Serum HMGB1 levels in EM patients were higher compared to the control group. Mig-SCog and HIT-6 scores were higher in the CM + MOH group compared to EM patients. HADS-A and HADS-D scores were significantly higher in the CM + MOH group compared to EM patients and healthy controls, and they were also higher in EM patients compared to healthy subjects. LPS levels were correlated with VE-cadherin and occludin levels. The number of monthly migraine headache days was positively correlated with serum LPS, HIF-1α, VE-cadherin, and IL-6 levels, HADS-A, HADS-D, HIT-6, and MigSCog scores.

**Conclusion:**

We have evidence for the first time that CM + MOH is associated with elevated serum LPS and LBP levels suggestive of LPS leak into the systemic circulation. Higher levels of nociceptive and/or pro-inflammatory molecules such as HMGB1, HIF-1α, IL-6, and CGRP may play a role in trigeminal sensitization and neurobiology of MOH. Intestinal hyperpermeability and consequent inflammatory response should be considered as a potential contributory factor in patients with MOH.

**Graphical Abstract:**

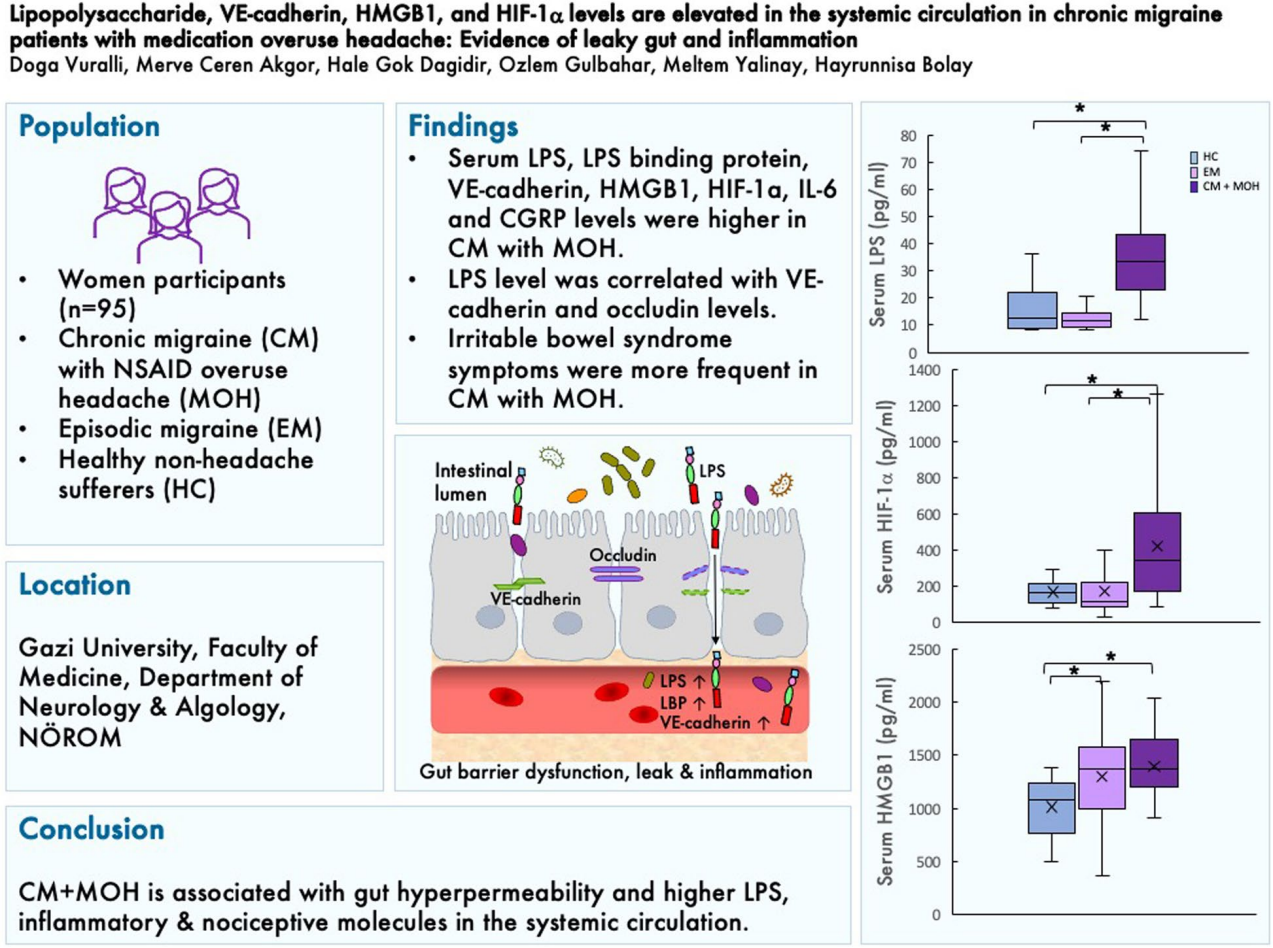

## Introduction

Chronic migraine (CM), a debilitating form of migraine, is associated with certain comorbidities such as depression, anxiety, fibromyalgia, and gastrointestinal disorders including irritable bowel syndrome (IBS). Medication overuse headache (MOH) is a secondary headache and is frequently associated with CM patients. Overuse of analgesic medications such as non-steroidal anti-inflammatory drugs (NSAIDs) for at least 3 months facilitates the development of MOH in migraine patients and NSAIDs are the most widely overused analgesic agents [1].

IBS is a functional gastrointestinal problem and is underdiagnosed in the population [2]. IBS differs from inflammatory bowel diseases such as Crohn’s Disease and ulcerative colitis in which a typical gastrointestinal inflammatory pathology is shown as an underlying mechanism. IBS is associated with changes in intestinal permeability and most of its symptoms are attributed to the presence of leaky gut. Disruption of intestinal and gastrovascular barriers leads to intestinal luminal contents leaking into the systemic circulation [3]. The lipopolysaccharide (LPS) is an important bacterial ingredient and must be kept in the intestinal compartment and has diverse effects depending on the amount present in the blood [4]. A large amount of LPS in the systemic circulation may lead to septic shock. However, when LPS leak into the bloodstream is limited as in the case of leaky gut, LPS acts as a proinflammatory agent [5].

Recently IBS symptoms were found to be more prevalent in patients with MOH compared to patients without MOH [1]. Long-term use of NSAIDs induces intestinal hyperpermeability, and leaky gut [6]. Routine doses of NSAIDs are shown to be toxic to human intestinal epithelium by disrupting mitochondrial oxidative phosphorylation and inducing oxidative stress [7]. NSAIDs can also facilitate the transport from the luminal to the basolateral compartment and disrupt tight junctions that are vital to keep LPS, a bacterial toxin of gram-negative bacteria, and other pathogenic molecules in the intestinal lumen [4, 7]. The intact intestinal barrier allows the passage of nutrients into the bloodstream whereas restricts the LPS leakage into the systemic circulation. Detection of LPS in the systemic circulation therefore indicates an impaired intestinal permeability and barrier function [3]. As a typical pathogen-associated molecular pattern, LPS in the bloodstream is a potent activator of inflammatory response in the host [5]. Increased levels of LPS have been reported in a variety of disorders. Tight junction proteins and adherens junction proteins are important in the intestinal integrity [8, 9], the gut-vascular barrier also serves as the last barrier to limit the passage of intestinal toxins into the blood circulation and a disruption in these structures may result in leaky gut syndrome [9, 10]. Lately, markers for increased intestinal permeability such as LPS, zonulin, and claudin were found to be elevated in the serum of major depression patients [11].

We recently showed elevated serum LPS binding protein (LBP) and tight junction protein occludin levels in addition to increased inflammatory response in two MOH models induced by different NSAIDs [4, 12]. Serum LBP, occludin levels, and brain high mobility group box-1 protein (HMGB1) and interleukin-17 (IL-17) levels were significantly increased in male rats that manifested headache behavior such as reduced periorbital pain thresholds, increased grooming, freezing and head shakes [12]. Serum LBP, IL-17, occludin, vascular endothelial cadherin (VE-cadherin), and calcitonin gene-related peptide (CGRP) levels, and brain HMGB1 and IL-17 levels were also found to be higher in another MOH model using oral piroxicam in female rats [4].

We hypothesized that MOH patients using NSAIDs have increased intestinal barrier permeability and higher LPS and inflammatory markers in the systemic circulation. CGRP is a fundamental neuropeptide in migraine neurobiology and increased in migraine attacks [13]. Interleukin 6 (IL-6) is a proinflammatory and nociceptive cytokine in the trigeminal system and was shown to be elevated in patients with migraine [14, 15]. IL-17 is implicated in both pre-clinical headache models [16] and inflammatory intestinal diseases. Innate immunity and damage-associated molecular pattern molecule HMGB1, and IL-6 were higher in COVID-19 headache patients and were correlated with headache severity and paracetamol unresponsiveness [17]. LBP is a dynamically regulated protein that binds to LPS and can be used as a marker to monitor LPS in circulation [18–20]. Tight junction (occludin) and adherens junction (VE-cadherin) proteins are core elements in the intestinal barrier and their detection in the bloodstream may indicate the disruption in intestinal permeability. Therefore, we aimed to measure the levels of the aforementioned molecules in CM patients with MOH and assess their correlations with the clinical features.

The presence of leaky gut in MOH has never been clinically investigated. We aimed to investigate whether CM patients with MOH have elevated LPS levels, markers associated with increased intestinal permeability, and inflammatory molecules in the systemic circulation. This study will provide information about the possible link between NSAID overuse headache and altered intestinal permeability.

## Methods

Female CM patients with MOH (NSAID overuse headache) and female episodic migraine (EM) patients were consecutively recruited from the headache outpatient clinic of Gazi University Faculty of Medicine, Department of Neurology and Algology between December 2022 and June 2023. Only women participants were included in the study. The other inclusion criteria for CM patients with MOH were; (1) age between 18-and 65 years of age, (2) having a definite diagnosis of chronic migraine (1.3) according to the International Classification of Headache Disorders 3rd edition (ICHD-3), 4) having a diagnosis of NSAID overuse headache (8.2.3.2) according to ICHD-3 with regular intake of ≥ 1 NSAID on ≥ 15 days/month for more than 3 months, 5) no use of any migraine preventive medications, 6) agreeing to give a blood sample for enzyme-linked immunosorbent assay analysis, and 7) having normal white blood cell count (WBC), eryhtrocyte sedimentation rate (ESR) and C-reactive protein (CRP) levels. Episodic migraine patients were included if, (1) they were between 18 and 65 years of age, (2) they had a definite diagnosis of migraine without aura (1.1) according to ICHD-3, (3) they were not using any preventive medications, (4) they agreed to give a blood sample for enzyme-linked immunosorbent assay analysis, and (5) they had normal WBC, ESR, and CRP levels. The healthy controls were included if, (1) they were between 18 and 65 years of age, (2) they had no history of headaches and (3) they had normal WBC, ESR, and CRP levels. Exclusion criteria for the participants were; (1) having any other chronic disease or neurological disease or any laboratory findings suggestive of chronic systemic disease (2) chronic daily use of any medications (3) any infections within 3 days before and after the blood sample collection 3) history of alcohol or drug abuse (4) smoking and (5) having any major psychiatric disease. The patients were included in the study by two headache experts (DV and HB) after a detailed clinical assessment. The blood samples for ELISA analysis were collected in the interictal period in the EM patients (headache-free for three days before and after the blood sample collection). The patients were called a month later to question whether they received a diagnosis of any chronic disease within one month after the blood sample collection.

The number of migraine headache days, migraine duration, migraine-related subjective cognitive symptoms scale (MigSCog) scores, and the Headache Impact Test (HIT-6) scores were recorded for both CM patients with MOH and EM patients. Hospital Anxiety and Depression Scale Anxiety (HADS-A) and Depression subscales (HADS-D) scores were recorded for all three groups. The presence of diagnostic irritable bowel syndrome symptoms was evaluated in all groups according to the Rome IV Criteria [21].

Serum LPS, LBP, CGRP, HMGB1, hypoxia-inducible factor-1α (HIF-1α), IL-17, IL-6, occludin, and VE-cadherin levels were assessed using enzyme-linked immunosorbent assay (ELISA). The blood samples collected from both the patients and the healthy controls were centrifuged (20 min 1000*g at 2–8 °C) to obtain serum samples according to the ELISA kit protocol. Serum samples were stored at − 80 °C until the analysis. The study was approved by the local ethics committee and was conducted according to the standards set by the Declaration of Helsinki.

### Enzyme-Linked Immuno Sorbent Assay (ELISA)

High-sensitivity ELISA kits were used to measure serum LPS, LBP, CGRP, HMGB1, HIF-1α, IL-17, IL-6, occludin, and VE-cadherin levels. ELISA kits were obtained from Elabscience Biotechnology Inc., Houston, TX, USA [Human LBP (E-EL-H6108), CGRP1 (E-EL-H0619), IL-17 (E-EL-H0105), IL-6 (E-EL-H6156), HMGB1 (E-EL-H1554), HIF-1 α (E-EL-H6066), Occludin (E-EL-H1073), VE-cadherin (E-EL-H6103)] and CUSABIO Inc., Wuhan, China [Human LPS (CSB-E0994h)]. The coefficients of variation for repeatability were < 10%.

All of the reagents were brought to room temperature before use. Reference standards provided in each kit were diluted according to the instructions of the manufacturer. Wash buffers were prepared by adding analytical-grade deionized water to the concentrated wash buffers. The kits were used according to the assay procedure. Combiwash Human ELISA plate washer was used for the washing processes. The enzyme-substrate reaction was terminated by adding a stop solution and a rapid color change to yellow was observed. Chromate microplate reader was used to measure the optical density of each well at 450 nm.

### Statistical analysis

Data analysis was performed using IBM SPSS statistical software version 22.0 (USA). The normal distribution of data was investigated by the Kolmogorov-Smirnov test. Continuous variables were indicated as mean ± standard deviation and categorical variables were given as frequency and percentage values. The analysis of normally distributed continuous variables was performed with one way-ANOVA when there were three groups and student t-test when there were two groups. Post-hoc analysis was performed using Šidák’s multiple comparisons test. The analysis of non-normally distributed continuous variables was performed with the Kruskal-Wallis test when there were three groups and Mann-Whitney’s U test when there were two groups. Post hoc analysis after the Kruskal-Wallis test was performed by Dunn’s multiple comparisons test. The analysis of categorical variables was performed using Pearson’s chi-square test. Spearman correlation analysis was performed to assess possible correlations between clinical features, HADS-A, HADS-D, Mig-SCog, HIT-6 scores, CGRP, and serum markers associated with leaky gut and inflammation. *p* < 0.05 was considered statistically significant.

## Results

**Fig. 1:**
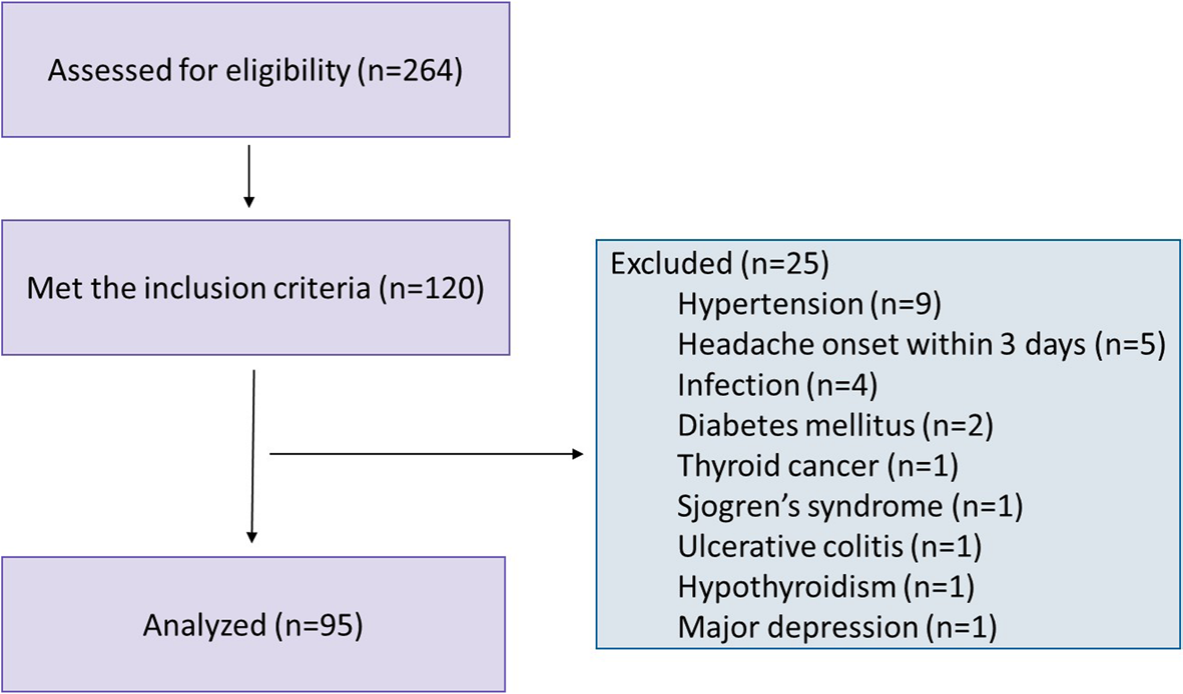
The flowchart of the study

The flowchart of the study is given in Fig. 1. Forty CM + MOH patients, 35 EM patients, and 20 healthy controls were included in the study. The mean age of the subjects was 38.0 ± 7.6 years in the chronic migraine and MOH group, 35.1 ± 7.6 years in the episodic migraine group, and 39.6 ± 11.1 years in the healthy control group (*p* = 0.14). Migraine duration was 233.8 ± 86.9 months in the chronic migraine and MOH group and 193.1 ± 93.6 months in the episodic migraine group (*p* = 0.061). Mean monthly migraine headache days was 20.5 ± 6.6 in the chronic migraine and MOH group and 2.7 ± 1.4 in the episodic migraine group (*p* < 0.0001).

**Fig. 2:**
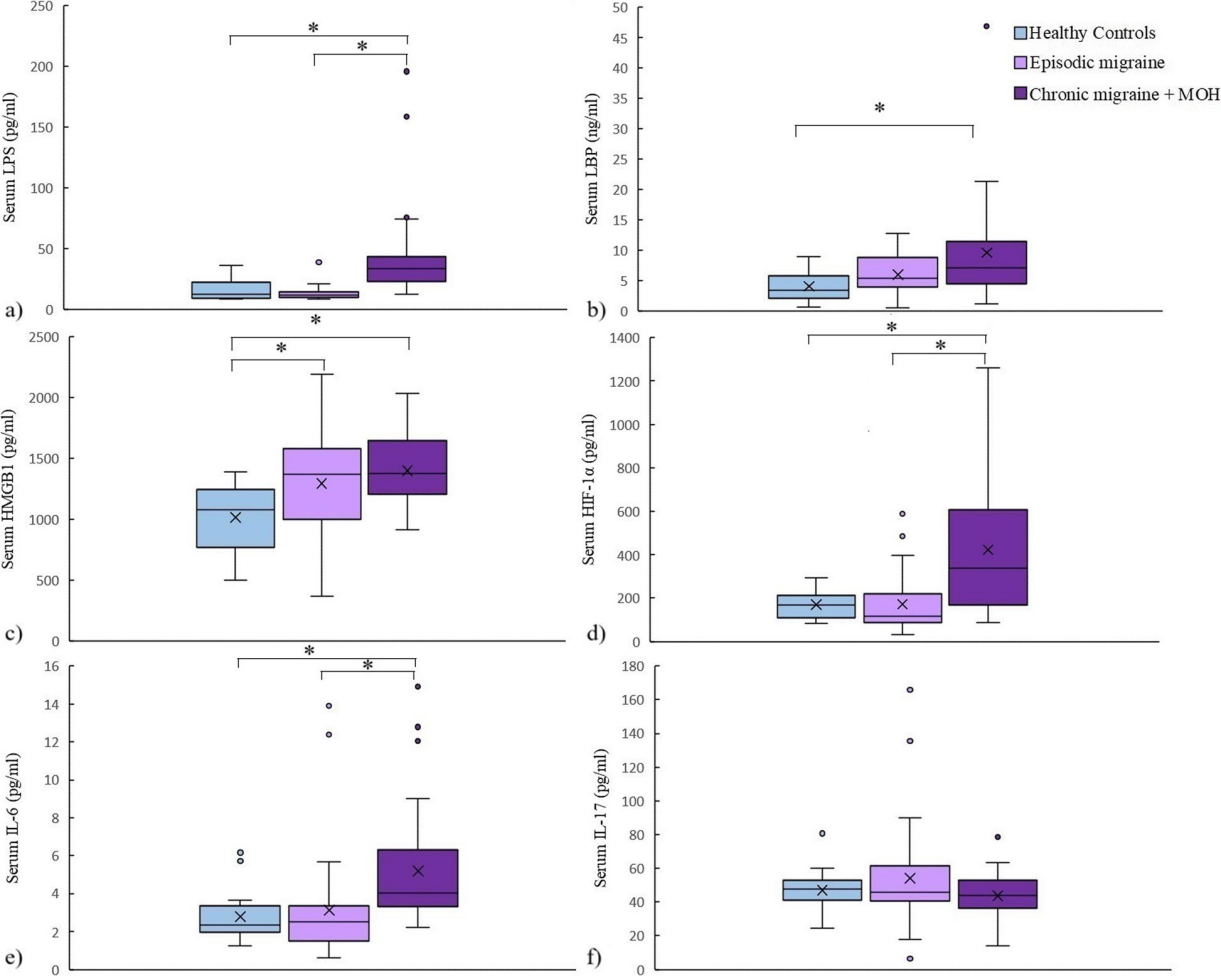
Data are shown as whisker-box plots (whisker: full range; line: median; cross: mean). **a** Serum LPS was higher in CM + MOH patients compared to EM patients (*p* < 0.0001) and healthy controls (*p* = 0.001), **b** serum LBP was higher in CM + MOH patients compared to healthy controls (*p* = 0.041), **c** serum HMGB1 was higher in CM + MOH patients (*p* = 0.0001) and EM patients (*p* = 0.014) compared to the healthy controls, **d** serum HIF-1α was higher in the CM + MOH patients compared to EM patients (*p* = 0.012) and healthy controls (*p* = 0.025), **e** serum IL-6 levels were higher in the CM + MOH patients compared to EM patients (*p* = 0.003) and healthy control (*p* = 0.005) and serum IL-17 levels were similar between the three groups (*p* = 0.101). Statistical analysis was performed with Kruskal-Wallis followed by post hoc Dunn’s multiple comparisons test for LPS, HIF-1α, and IL-6 data. Statistical analysis was performed with one way-ANOVA followed by post-hoc Šidák’s test for multiple comparisons when required for all other data. * *p* < 0.05

**Fig. 3:**
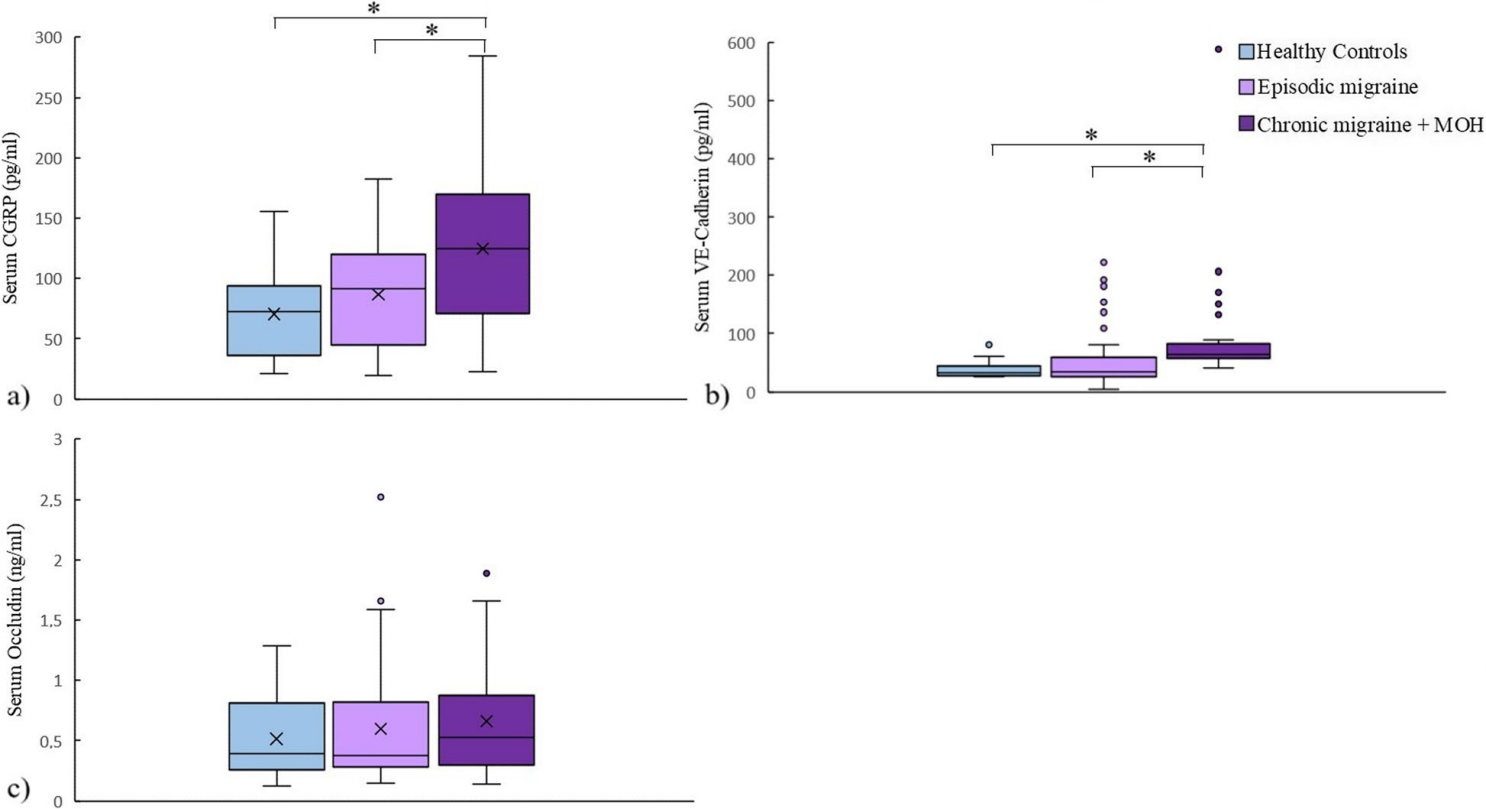
Data are shown as whisker-box plots (whisker: full range; line: median; cross: mean). **a** Serum CGRP was higher in the CM + MOH patients compared to EM patients (*p* = 0.005) and healthy controls (*p* = 0.025), **b** serum VE-cadherin levels were higher in the CM + MOH patients compared to the EM patients (*p* < 0.0001) and healthy controls (*p* < 0.002) and (**c**) serum occludin levels were comparable between the groups (*p* = 0.47). Statistical analysis was performed with Kruskal-Wallis followed by post hoc Dunn’s multiple comparisons test. * *p* < 0.05

**Fig. 4:**
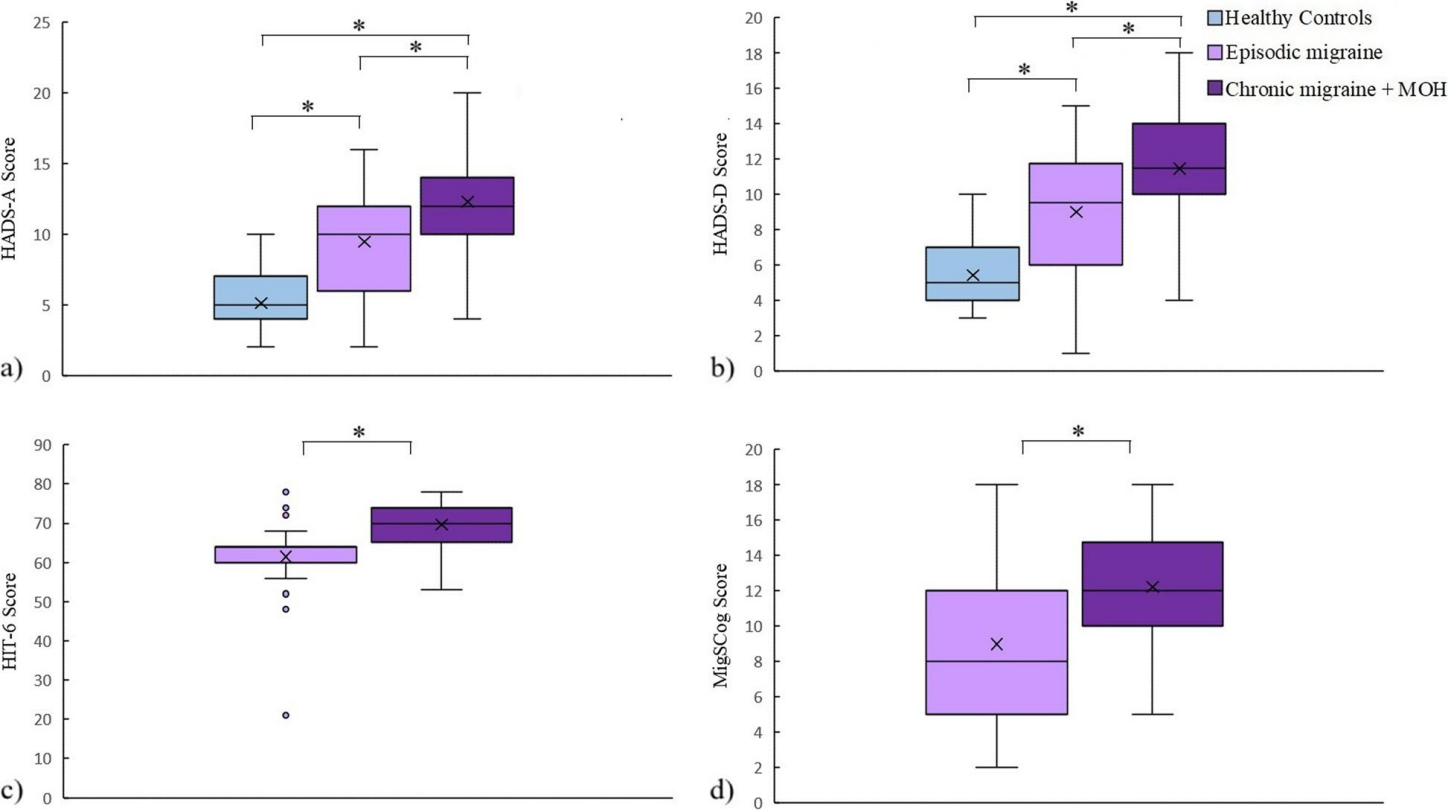
Data are shown as whisker-box plots (whisker: full range; line: median; cross: mean). **a** CM + MOH patients had significantly higher HADS-A scores compared to EM patients (*p* = 0.002) and healthy controls (*p* < 0.0001) and HADS-A scores were also higher in EM patients compared to healthy controls (*p* < 0.0001), **b** CM + MOH patients had significantly higher HADS-D scores compared to EM patients (*p* = 0.007) and healthy controls (*p* < 0.0001) and HADS-D scores were also higher in EM patients compared to healthy controls (*p* = 0.001) (**c**) HIT-6 scores were significantly higher in the CM + MOH group compared to the EM group (*p* < 0.0001) and (**d**) MigSCog scores were significantly higher in the CM + MOH patients compared to EM patients (*p* < 0.0001). Statistical analysis was performed with one way-ANOVA followed by post-hoc Šidák’s test for multiple comparisons for HADS-A and HADS-D. Data analysis was performed with Mann-Whitney’s U test for HIT-6 scores and student t-test for MigSCog scores. * *p* < 0.05

**Fig. 5:**
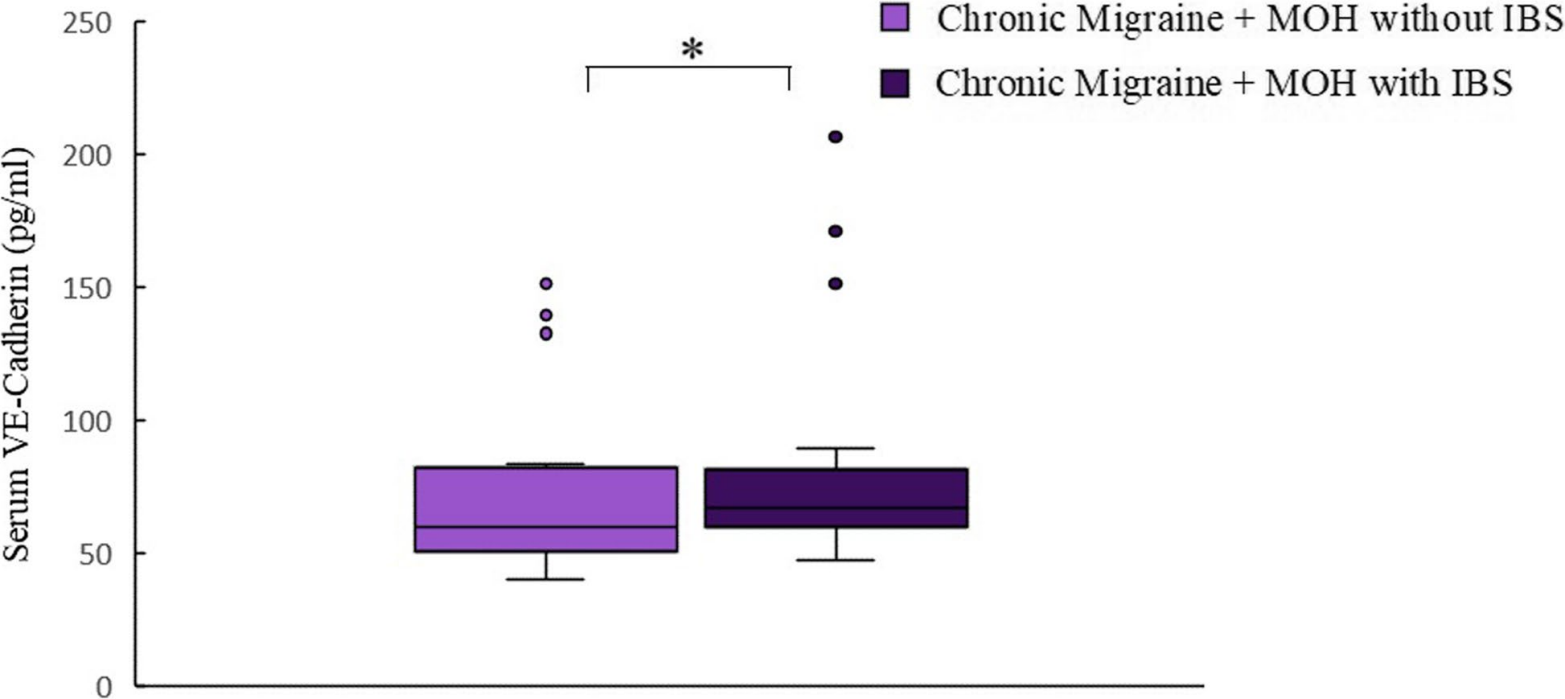
Data are shown as whisker-box plots (whisker: full range; line: median). CM + MOH patients with IBS had significantly higher serum VE-cadherin levels compared to CM + MOH patients without IBS (*p* = 0.045). Data analysis was performed with Mann-Whitney’s U test. * *p* < 0.05

Serum LPS was significantly different between the three groups (*p* < 0.0001). Serum LPS was significantly higher in the CM + MOH patients compared to EM patients and healthy controls (*p* < 0.0001 and *p* = 0.001, respectively). Serum LBP was significantly higher in the CM + MOH patients compared to healthy controls (*p* = 0.041) (Fig. 2). Serum HMGB1 was significantly different between the three groups (*p* = 0.001). HMGB1 was significantly higher in CM + MOH patients (*p* = 0.0001) and EM patients (*p* = 0.014) compared to the healthy controls (Fig. 2). Serum HIF-1α was significantly different between the groups (*p* = 0.014). Serum HIF-1α was significantly higher in the CM + MOH patients compared to EM patients and healthy controls (*p* = 0.012 and *p* = 0.025 respectively) (Fig. 2). Serum IL-6 levels were significantly different between the groups (*p* = 0.002). Serum IL-6 levels were higher in the CM + MOH patients compared to EM patients and healthy controls (*p* = 0.003 and *p* = 0.005 respectively) (Fig. 2). Serum IL-17 levels were comparable between the three groups (*p* = 0.101) (Fig. 2). Serum CGRP was significantly different among the groups (*p* = 0.008). CGRP was higher in the CM + MOH patients compared to EM patients and healthy controls (*p* = 0.005 and p = 0.025 respectively) (Fig. 3). Serum VE-cadherin levels were significantly different between the groups (*p* < 0.0001). Serum VE-cadherin levels were higher in the CM + MOH patients compared to the EM patients (*p* < 0.0001) and healthy controls (*p* < 0.002) (Fig. 3). Serum occludin levels were similar between the three groups (*p* = 0.47) (Fig. 3). HADS-A scores were significantly different between the three groups (*p* < 0.0001). HADS-A scores were significantly higher in the CM + MOH patients compared to EM patients (*p* = 0.002) and healthy controls (*p* < 0.0001) (Fig. 4). HADS-A scores were also significantly higher in EM patients compared to healthy controls (*p* < 0.0001) (Fig. 4). HADS-D scores were significantly different between the three groups (*p* < 0.0001). HADS-D scores were significantly higher in the CM + MOH patients compared to EM patients (*p* = 0.007) and healthy controls (*p* < 0.0001) (Fig. 4). HADS-D scores were also significantly higher in EM patients compared to healthy controls (*p* = 0.001) (Fig. 4). HIT-6 scores were significantly higher in the CM + MOH group compared to the EM group (*p* < 0.0001) (Fig. 4). MigSCog scores were significantly higher in the CM + MOH patients compared to EM patients (*p* < 0.0001) (Fig. 4). IBS symptoms were defined by 19 CM + MOH patients (47.5%), 11 EM patients (31.4%), and 5 healthy controls (25%). IBS symptoms were higher in CM + MOH patients (*p* = 0.0018). Serum VE-cadherin levels in CM + MOH patients with IBS were higher than in CM + MOH patients without IBS (*p* = 0.045) (Fig. 5). Other serum markers were comparable between CM + MOH patients with IBS or without IBS.

Correlation Analysis.

The number of monthly migraine headache days was positively correlated with serum LPS (*r* = 0.512, *p* < 0.001), HIF-1α (*r* = 0.622, *p* < 0.0001), VE-cadherin (*r* = 0.437, *p* < 0.0001), IL-6 levels (*r* = 0.474, *p* < 0.0001), migraine duration (*r* = 0.676, *p* < 0.0001), HADS-A (*r* = 0.439, *p* < 0.0001), HADS-D (*r* = 0.417, *p* < 0.0001), HIT-6 (*r* = 0.513, *p* < 0.0001) and MigSCog scores (*r* = 0.424, *p* < 0.0001) in CM patients with MOH and EM patients. Serum LPS levels were positively correlated with VE-cadherin levels (*r* = 0.450, *p* = 0.001) and occludin levels (*r* = 0.497, *p* < 0.0001) in all groups. Serum IL-6 levels were positively correlated with serum occludin levels (*r* = 0.550, *p* < 0.0001) and serum CGRP levels (*r* = 0.494, *p* < 0.0001) in all groups. Migraine duration was positively correlated with serum HIF-1α (*r* = 0.712, *p* < 0.0001), and IL-6 levels (*r* = 0.476, *p* < 0.0001), HADS-A (*r* = 0.554, *p* < 0.0001), HADS-D (*r* = 0.452, *p* < 0.0001), and HIT-6 scores (*r* = 0.505, *p* < 0.0001) in CM patients with MOH and EM patients. Serum HIF-1α was also positively correlated with HADS-A (*r* = 0.430, *p* < 0.0001) and HADS-D scores (*r* = 0.550, *p* < 0.0001). HADS-A scores were also positively correlated with HADS-D scores (*r* = 0.690, *p* < 0.0001) in all three groups.

## Discussion

Our study provided strong evidence for leaky gut and inflammatory response in the systemic circulation in CM patients with MOH. Higher serum LPS and LBP levels indicated an increased passage of LPS into the bloodstream due to intestinal hyperpermeability. Serum levels of adherens junction protein VE-cadherin were increased in CM patients with MOH. VE-cadherin is a biomarker for gut-vascular barrier integrity and increased soluble VE-cadherin is also related to leaky gut in CM patients with MOH. VE-cadherin levels were higher in CM + MOH patients with IBS symptoms compared to CM + MOH patients without IBS and serum LPS levels were correlated with serum VE-cadherin and occludin levels which also supported leaky gut.

Intestinal hyperpermeability in CM patients with MOH was associated with an inflammatory response as serum levels of HMGB1, HIF-1α, IL-6, and CGRP were elevated. Although occludin levels were not significantly altered among the three groups, occludin levels were positively correlated with LPS and IL-6 levels. The number of monthly migraine headache days was correlated with serum levels of LPS, HIF-1α, VE-cadherin, and IL-6. An increase in intestinal permeability and related inflammation was associated with higher migraine frequency. Our novel study indicated that increased intestinal permeability and consequent nociceptive and/or inflammatory response could be a potential underlying factor in the neurobiology of NSAID overuse headache.

The possible role of altered gut permeability and related inflammation in patients with MOH has not been investigated before. Even though MOH is commonly seen in migraine patients and various studies have focused on proinflammatory cytokines in migraine, a link between inflammation and leaky gut has not been investigated in MOH patients. In a recent meta-analysis, IL-6, IL-8, IL-1β, and tumor necrosis factor α were the proinflammatory cytokines implicated in migraine patients [22]. The role of the gut-brain axis in migraine headache has already been suggested in several papers [23] and different gut microbiome profiles were reported in episodic and chronic migraine patients, and healthy controls [24].

Translational studies investigated the intestinal permeability change in NSAID-induced MOH models [4, 12]. In MOH rodent models both in female and male rats with different NSAIDs, elevated serum LBP and occludin levels and increased brain HMGB1 and IL-17 levels were reported as evidence of leaky gut and associated inflammation in the brain respectively [4, 12]. Moreover, serum VE-cadherin, IL-17, and IL-6 were also higher in the MOH model using piroxicam in female rats supporting intestinal hyperpermeability and systemic inflammation in MOH [4].

IBS is a functional bowel disorder, manifesting with abdominal pain, bloating, and altered bowel habits, which is not explained by any other structural or inflammatory disease. IBS is diagnosed based on the presence of specific gastrointestinal symptoms and IBS constitutes 20–50% of the gastroenterology referrals [25]. The prevalence of IBS is reported to differ between 11 and 31% [26]. MOH patients have higher IBS symptoms [1] which is associated with intestinal barrier dysfunction [27]. IBS is an under-diagnosed entity in migraine and MOH patients and recently in a cross-sectional survey study, 44.3% of episodic migraine patients and 65% of the MOH patients had IBS symptoms while 35% of the participants without headaches had IBS according to Rome-IV criteria [1]. The frequencies of IBS symptoms in our study were, 47.5%, 31.4%, and 25% in CM patients with MOH, EM patients, and healthy controls respectively. IBS is more frequent in women [28] and the higher frequencies of IBS in this study could be because our study population only consisted of women subjects. Moreover, publications are reporting more than 75% of the patients with IBS symptoms remain undiagnosed in the population [2, 28].

In this study, serum IL-6 levels were higher in CM patients with MOH compared to EM patients and healthy controls whereas no significant alterations were shown in serum IL-17 levels among the three groups. IL-6 is one of the key pro-inflammatory molecules in the central nervous system (CNS) and implicated in migraine disease in a recent meta-analysis [29]. The studies on IL-6 levels in the literature on migraine have reported inconclusive results. Some studies reported higher IL-6 levels in migraine while others showed similar IL-6 levels in both migraine patients and healthy controls [29]. In a recent study, the correlation between higher serum IL-6 levels and migraine chronification has been reported [14]. Similarly, in this study, serum IL-6 levels were found to be higher in CM patients with MOH compared to EM patients and healthy subjects. Serum IL-17 levels were not investigated in migraine patients previously. In rats, higher brain IL-17 levels were shown in a preclinical nitroglycerin migraine model [16] and two NSAID-induced MOH models [4, 12]. Increased serum IL-17 levels were also shown in one of the latter MOH models induced by piroxicam [4]. However, serum IL-17 levels in CM patients with MOH and EM patients were comparable to healthy controls in our study.

CGRP levels were significantly elevated in CM patients with MOH compared to EM patients and healthy controls. Higher circulating CGRP was suggested as a biomarker for chronic migraine [30]. Increased serum CGRP levels may not only indicate chronic migraine headache but also may have a role in the maintenance of trigeminal nociception. Serum CGRP levels were correlated with serum IL-6 levels similar to a previous study in which CGRP levels were correlated with IL-6 levels during migraine attacks [31].

We found elevated HMGB1 serum levels in CM patients with MOH and EM patients compared to healthy controls suggesting that HMGB1 could be a marker for migraine rather than chronicity. Further studies in larger populations will elucidate the role of HMGB1 in migraine and MOH. Increased serum HMGB1 could be related to inflammation induced both by intestinal hyperpermeability and headache pathophysiology in migraine patients. The relation between HMGB1 and intestinal barrier function is more complicated. HMGB1 may further increase the intestinal damage caused by NSAIDs [32] and may result in higher intestinal permeability and augmented inflammatory response in CM patients with MOH. Even though HMGB1 levels were comparable between CM patients with MOH and EM patients, the impact of HMGB1 could be higher in CM patients with MOH. HMGB1 was shown to be increased in the cerebral cortex in translational MOH models in both female and male rats [4, 12]. Elevated serum HMGB1 was also previously reported in the COVID-19 headache [17]. HMGB1 and LPS act on the same receptor TLR-4 [33, 34], which is widely expressed in trigeminal neurons and nerve fibers and modulates innate immune reactions. Higher levels of both LPS and HMGB1 in the systemic circulation denote ongoing inflammation and probably contribute to nociception.

A low brain uptake of circulating LPS was shown in a murine BBB model [35] and brain entrance of LPS possibly via lipoprotein-mediated transport was suggested in a rat model [36]. Recently in an animal model using CD-1 male mice, bidirectional passage of HMGB1 across the blood brain barrier (BBB) has been shown [37]. Entrance of LPS and HMGB1 into CNS in migraine patients would contribute to sustained central sensitization, neuroinflammation, migraine chronification and poor response to treatments. However, there is no direct evidence for the passage of either LPS or HMGB1 into CNS in humans.

HIF-1α regulates mitochondrial function and metabolism, and adaptation to hypoxic conditions. Migraine attacks were recently shown to be accompanied by elevated HIF-1α, FGF-21, GDF-15, CGRP, and PACAP-38 in medication-naïve children with migraine [38]. HIF-1α levels were elevated in CM patients with MOH compared to EM patients and healthy controls in our study. An increase in HIF-1α may be associated with mitochondrial distress and inflammation in chronic migraine [38]. Stimulation of primary glial and meningeal cell cultures with nitroglycerin, a headache trigger in migraineurs, was shown to alter intracellular iron trafficking which might have an impact on oxidative stress [39]. Iron deposition correlated with headache frequency and duration was shown in subcortical brain structures in migraine patients [40, 41]. The correlations between serum HIF-1α levels and the number of migraine headache days/month, migraine duration, HADS-A and HADS-D scores, suggest that elevated serum HIF-1α levels are associated with increased migraine headache frequency, migraine chronification, and higher anxiety and depression scores.

A wide range of inflammatory responses were shown in the central nervous system and peripheral trigeminal nociceptive pathways in translational migraine models. Moreover, altered systemic pro-inflammatory and anti-inflammatory cytokine levels were demonstrated in migraine patients [29]. Even though neuro-inflammation and systemic inflammatory reactions are common findings, the driving sources of this inflammation are not clarified yet. We suggest leaky gut and consequent LPS escape to the bloodstream could be one of the sources of systemic inflammation. Additionally, elevated pro-inflammatory molecules such as LPS, HMGB1, and IL-6 may exert a synergistic effect on the trigeminal neurons with their nociceptive properties.

How increased intestinal permeability would affect response to anti-CGRP monoclonal antibodies (CGRP mAbs) is unknown and remains to be investigated. Even though there is not enough evidence regarding this matter, anti-CGRP mAbs may impede with neuron-goblet cell signaling via CGRP, leading to disruption of the intestinal barrier protection [42] and the intestinal barrier function. Therefore, it is plausible that the interference of mAbs with CGRP in the gut may worsen intestinal leak and contribute to the unresponsiveness towards anti-CGRP mAbs treatment.

Migraine influences cognitive functions in multiple domains such as attention, memory, processing speed, executive functions, and verbal skills [43]. Migraine patients frequently complain of cognitive problems interfering with daily life. MigSCog quantifies subjective cognitive complaints of migraine patients. In this study, we showed higher MigSCog scores in CM patients with MOH compared to EM patients. MigSCog scores were positively correlated with the number of monthly migraine headache days showing that subjective cognitive symptoms increase with migraine chronification.

The strengths of the study are as follows; (1) we showed the presence of leaky gut in CM patients with NSAID overuse headache with increased LPS, LBP, and VE-cadherin levels in the bloodstream and the correlation of LPS levels with VE-cadherin and occludin levels (2) LPS, HMGB1, HIF-1α, IL-6 and CGRP levels were elevated indicating heightened innate immunity, proinflammatory and nociceptive response, (3) patients were examined and included in the study by headache specialist neurologists, (4) all of the subjects were women and MOH patients overusing NSAIDs were included, which increase the homogeneity of the study population as MOH is more prevalent in women and NSAIDs are the most commonly used analgesics. The limitations of the study are that (1) the size of the study population is small, (2) CM patients without MOH are not included, (3) detailed objective cognitive testing is not performed, and (4) gut microbial profiles were not studied. We did not include CM patients overusing triptans or other analgesics in this study population. The preliminary findings of this study should be confirmed in larger patient populations. Also the presence of leaky gut in patients overusing analgesics other than NSAIDs remains to be explored.

## Conclusion

The study showed for the first time that in CM patients with MOH, circulating levels of LPS, LBP, and VE-cadherin were elevated indicating an increased passage of LPS into the bloodstream. Also, the frequency of IBS was higher in CM patients with MOH. Higher levels of nociceptive and/or proinflammatory molecules such as HMGB1, HIF-1α, IL-6, and CGRP may play a role in trigeminal sensitization and pathophysiology of MOH. Intestinal hyperpermeability and consequent inflammatory response should be considered as a potential contributor in CM patients with NSAID overuse headache. Future studies will determine the causal relation and possible targets to interfere with MOH and leaky gut.

## Data Availability

The data that support the findings of this study are available from the corresponding author upon reasonable request.

## Abbreviations

CM: Chronic migraine
IBS: Irritable bowel syndrome
MOH: Medication overuse headache
NSAIDs: Non-steroidal anti-inflammatory drugs
LPS: Lipopolysaccharide
LBP: LPS binding protein
HMGB1: High mobility group box-1 protein
IL-17: Interleukin-17
VE-cadherin: Vascular endothelial cadherin
CGRP: Calcitonin gene-related peptide
IL-6: Interleukin 6
EM: Episodic migraine
WBC: White blood cell count
ESR: Eryhtrocyte sedimentation rate
CRP: C-reactive protein
ICHD-3: International Classification of Headache Disorders 3rd edition
MigSCog: Migraine-related subjective cognitive symptoms scale
HIT-6: Headache Impact Test
HADS-A: Hospital Anxiety and Depression Scale Anxiety subscale
HADS-D: Hospital Anxiety and Depression Scale Depression subscale
ELISA: Enzyme-linked immunosorbent assay
HIF-1α: Hypoxia-inducible factor-1α
CNS: Central nervous system
BBB: Blood brain barrier
